# A cautionary tale of hyponatraemia in an older person: Could This Have Been Prevented?

**DOI:** 10.51866/tyk.502

**Published:** 2025-07-22

**Authors:** Yi Bin Ho, Rebecca Kai Jan Choong, Kit Mun Tan

**Affiliations:** 1 MBBCh, FRCP, Geriatric Medicine Unit, Department of Medicine, Faculty of Medicine, Universiti Malaya, Kuala Lumpur, Malaysia. Email: kmtan@ummc.edu.my; 2 MD, MRCP, Geriatric Medicine Unit, Department of Medicine, Universiti Malaya Medical Centre, Lembah Pantai, Kuala Lumpur, Malaysia.; 3 MBBS, MRCP, Geriatric Medicine Unit, Department of Medicine, Faculty of Medicine, Universiti Malaya, Kuala Lumpur, Malaysia.

**Keywords:** Hyponatraemia, Hydrochlorothiazide, Carbamazepine, Drug interaction

## Abstract

Hyponatraemia is an exceedingly common condition frequently encountered both in hospital and primary care settings. This medical condition poses unique challenges due to its often subtle and nonspecific symptoms, alongside its diverse array of potential causes. It can result in serious consequences when overlooked or not treated appropriately. Herein, we illustrate the case of a 68-year-old woman who presented with symptomatic hyponatraemia. Although not uncommon, the cause of hyponatraemia in this patient is frequently overlooked but, importantly, is avoidable. This case underlines the necessity of a more watchful approach to prescription of medications, especially in older adults with multiple comorbidities.

## Case summary

We report the case of a 68-year-old woman with underlying diabetes mellitus, hypertension and focal epilepsy. Her epilepsy was well-controlled with carbamazepine (sustained release formulation) 400 mg twice daily for many years. She was previously on amlodipine 10 mg once daily for hypertension.

In September 2021, she complained of intermittent headaches that started from the occipital area, became generalised and were associated with dizziness. She visited her family doctor, who noticed that her blood pressure control was inadequate (systolic blood pressure: 140-160 mmHg). Hence, amlodipine was switched to losartan 100 mg OD and hydrochlorothiazide 25 mg OD in November 2021. Her blood pressure control improved (103-150/60-88 mmHg), and her headaches became less frequent. However, she complained of worsening dizziness and an unsteady gait. She also had frequent falls due to such dizziness. The dizziness was not a room-spinning sensation (not vertiginous) and not associated with posture change. She did not seek further medical attention during that time.

In February 2022, her family doctor arranged a routine blood test, which revealed hyponatraemia (serum sodium level: 121 mmol/L). Her oral intake was normal, and there was neither vomiting nor diarrhoea. She was referred to our emergency department and admitted for symptomatic hyponatraemia, with ongoing dizziness,thought to be exacerbated by the hyponatraemia. Clinically, she was euvolemic with normal power, tone and reflexes of all limbs. There were no cerebellar signs, and cranial nerve examination demonstrated unremarkable findings. The renal function and initial findings are shown in Table 1. Her brain computed tomography showed bilateral lentiform nuclei lacunar infarcts with no evidence of cerebral oedema.

**Table 1:** Blood investigation results upon presentation

	Normal value	25/2/2022	26/2/2022
Sodium (mmol/L)	136-145	119	
Potassium (mmol/L)	3.6-5.2	3.4	
Urea (mmol/L)	3.2-8.2	5.1	
Creatinine (μmol/L)	44-71	52	

Serum osmolality (mmol/kg)	275-295	260	
Urine osmolality (mmol/kg)	50-1200	355	
Urine sodium (mmol/L)	40-220	52	

TSH (mIU/L)	0.55-4.78	1.5	
Free T4 (pmol/L)	11.5-22.7	14.7	
Early morning cortisol (nmol/L)	145-619		614
Creatinine (μmol/L)	44-71	52	

Total cholesterol (mmol/L)	<5.2		4.7
Triglyceride (mmol/L)	<1.7		0.5
HDL (mmol/L)	>1.1		2.81
LDL (mmol/L)	<3.4		1.66
HbAlc (%)	<6.5		6.9

TSH: Thyroid Stimulating Hormone, Free T4: free thyroxine, HDL: high-density lipoprotein, LDL: low-density lipoprotein, HbAlc: Hemoglobin A1C

**Questions:**

1. What is the most likely cause of hyponatraemia in this patient?
2. What is the management?
3. How can this condition be avoided?

**Answers**

1. The most likely causes of hyponatraemia in this patient were hydrochlorothiazide and carbamazepine, and their combination likely led to a drug-drug interaction, additively worsening hyponatraemia.^12^
2. During the patient’s admission, her losartan and hydrochlorothiazide were stopped, and perindopril 4 mg OD commenced, while her carbamazepine was changed to levetiracetam 250 mg BD. The sodium level gradually improved, as shown in Table 2. Her headache and giddiness also gradually improved, and the patient was discharged well.
3. A meticulous medication review and diligent assessment for potential drug-drug interactions prior to prescription of a new medication especially in older persons are essential. Given the increased risk of adverse reactions in older adults, regular monitoring should be conducted after the initiation of new medications. This should include both symptom assessment and periodic blood tests to detect early signs of issues such as hyponatraemia or other electrolyte imbalances.

**Table 2:** Renal profile trend.

	Normal value	25/2/22 11 p.m.	26/2/22 2 p.m.	26/2/22 10 p.m.	27/2/22 5 a.m.	28/2/22 1 p.m.
Sodium (mmol/L)	136-145	119	121	126	128	130
Potassium (mmol/L)	3.6-5.2	3.4	3.6	4.3	3.4	3.7
Urea (mmol/L)	3.2-8.2	5.1	3.9	3.1	3.0	3.6
Creatinine (μmol/L)	44-71	52	43	41	41	45

## Discussion

Both thiazide diuretics and carbamazepine are independently associated with a risk of hyponatraemia. The mechanism of thiazide-induced hyponatraemia is thought to involve the inhibition of sodium reabsorption at the distal renal tubules via blockade of the thiazidesensitive sodium-chloride cotransporter (NCC). This impairs electrolyte transport in the diluting segment of the nephron and may reduce urinary dilution capacity in vulnerable populations.^3^ Female sex and older age are associated with a higher risk of hyponatraemia while on hydrochlorothiazide.^4^

The underlying reason for women’s increased susceptibility to the effects of thiazide diuretics remains unclear. One possible explanation is the greater expression of the thiazide-sensitive NCC in women, as demonstrated in animal studies.^5^ The inhibition of the NCC by thiazides may therefore lead to greater sodium loss and more pronounced volume depletion in women.^6^

Older persons taking thiazides may have a greater impairment in water excretion following a water load compared to younger individuals. This impaired urinary dilution capacity increases their susceptibility to thiazide-induced hyponatraemia.^3^

The mechanism by which carbamazepine causes hyponatraemia is thought to be potentiation of the actions of the antidiuretic hormone leading to dilutional hyponatraemia.^7^

Both carbamazepine and diuretics are identified in the Beers criteria as potentially inappropriate medications to be used with caution in older persons due to the potential to cause or exacerbate syndrome of inappropriate antidiuretic hormone secretion or hyponatraemia.^8^

As older patients have multiple underlying comorbidities and age-related physiological changes, such as reduced renal function, lower total body water, an impaired thirst mechanism and decreased regulation of sodium and water balance,^9^ even a minimal change in electrolytes can lead to morbidities and avoidable mortality.

Carbamazepine and hydrochlorothiazide have been reported to interact with each other and cause more severe hyponatraemia,^1,2^ but there are limited cases reported. This case illustrates an additive effect between hydrochlorothiazide and carbamazepine to cause hyponatraemia. This potential effect should be considered when prescribing anti-epileptics to patients receiving hydrochlorothiazide or diuretics to patients receiving carbamazepine.^8^ Review of preexisting medications before any adjustment of medications is essential to avoid potential drug interactions. This can be performed using online drug interaction checkers, including the Medscape website (https://reference.medscape.com/drug-interactionchecker), which is free of charge at present.

Since diuretics such as hydrochlorothiazide are a known cause of hyponatraemia, especially in older patients with a history of diuretic-induced hyponatraemia, it would be advisable to avoid them.^10^ Instead, alternative antihypertensive agents such as calcium channel blockers, angiotensin-converting enzyme inhibitors or angiotensin II receptor blockers can be used. If diuretics are chosen, a lower dose should be preferred, and regular monitoring is essential after the initiation. This includes symptom assessment and periodic blood tests to detect early signs of issues such as hyponatraemia or other electrolyte imbalances.^6,10^

Hyponatraemia is a common condition frequently encountered both in hospital and primary care settings. In the hospital, treatment and management of hyponatraemia includes stopping the offending medications and administration of intravenous fluids such as normal saline. Hypertonic saline is very rarely required, usually in cases of markedly severe hyponatraemia where the person is at risk of or has neuorological symptoms. Rapid increase of sodium levels should be avoided to reduce the risk of osmotic demyelination syndrome.^11^ In primary care, hyponatraemia is often identified during routine monitoring for chronic conditions, prompting a focused re-evaluation to identify underlying causes. Management typically emphasises longterm strategies, including medication review, lifestyle modifications and gradual titration of blood pressure medications.^12^

## Conclusion

This case emphasises the importance of a thorough medication review and check for potential drug-drug interactions prior to prescription of a new medication in older persons.
